# Statistical Design of Experimental and Bootstrap Neural Network Modelling Approach for Thermoseparating Aqueous Two-Phase Extraction of Polyhydroxyalkanoates

**DOI:** 10.3390/polym10020132

**Published:** 2018-01-30

**Authors:** Yoong Kit Leong, Chih-Kai Chang, Senthil Kumar Arumugasamy, John Chi-Wei Lan, Hwei-San Loh, Dinie Muhammad, Pau Loke Show

**Affiliations:** 1 Bioseparation Research Group, Department of Chemical and Environmental Engineering, Faculty of Engineering, University of Nottingham Malaysia Campus, Jalan Broga, 43500 Semenyih, Selangor Darul Ehsan, Malaysia; yoongkitleong1014@gmail.com; 2 Biorefinery and Bioprocess Engineering Laboratory, Department of Chemical Engineering and Materials Science, Yuan Ze University, No. 135 Yuan-Tung Road, Chung-Li, Tao-Yuan 32003, Taiwan; kevinvecent@gmail.com; 3 Department of Chemical and Environmental Engineering, Faculty of Engineering, University of Nottingham Malaysia Campus, Jalan Broga, 43500 Semenyih, Selangor Darul Ehsan, Malaysia; senthil.arumugasamy@nottingham.edu.my; 4 School of Biosciences, Faculty of Science, University of Nottingham Malaysia Campus, Jalan Broga, 43500 Semenyih, Selangor Darul Ehsan, Malaysia; sandy.loh@nottingham.edu.my; 5 School of Chemical Engineering, University Sains Malaysia, Engineering Campus, Seri Ampangan, 14300 Nibong Tebal, Seberang Perai Selatan, Pulau Pinang, Malaysia; annursi@gmail.com

**Keywords:** aqueous two-phase extraction, bioseparations, design of experiment, purification, polyhydroxyalkanoates

## Abstract

At present, polyhydroxyalkanoates (PHAs) have been considered as a promising alternative to conventional plastics due to their diverse variability in structure and rapid biodegradation. To ensure cost competitiveness in the market, thermoseparating aqueous two-phase extraction (ATPE) with the advantages of being mild and environmental-friendly was suggested as the primary isolation and purification tool for PHAs. Utilizing two-level full factorial design, this work studied the influence and interaction between four independent variables on the partitioning behavior of PHAs. Based on the experimental results, feed forward neural network (FFNN) was used to develop an empirical model of PHAs based on the ATPE thermoseparating input-output parameter. In this case, bootstrap resampling technique was used to generate more data. At the conditions of 15 wt % phosphate salt, 18 wt % ethylene oxide–propylene oxide (EOPO), and pH 10 without the addition of NaCl, the purification and recovery of PHAs achieved a highest yield of 93.9%. Overall, the statistical analysis demonstrated that the phosphate concentration and thermoseparating polymer concentration were the most significant parameters due to their individual influence and synergistic interaction between them on all the response variables. The final results of the FFNN model showed the ability of the model to seamlessly generalize the relationship between the input–output of the process.

## 1. Introduction

Conventional plastics have become an indispensable part of human daily life owing to their wide range of applications [1]. Nevertheless, governments and industries are pouring effort into seeking biodegradable, renewable, and economical substitutes to replace petrochemical-based plastics, driven by the rising environmental awareness towards plastic pollution and the rapidly depleting crude oil reserve [2]. Polyhydroxyalkanoates (PHAs) stand out among different types of biodegradable polyesters like cellulosic polymers, polylactic acid, starch derivatives and others due to their distinctive properties of wide variability in structure and rapid biodegradation without the need of a special environment (3–9 months) [3]. PHAs are thermoplastics with the attractive characteristics of being renewable, biodegradable, biocompatible, non-toxic, inert, water-insoluble, indefinitely stable in air, and having properties similar to conventional plastics [4,5,6]. Therefore, PHAs have an extensive range of applications for disposable utensils, packaging, machinery housing, and accessories manufacturing as well as huge potential in the medical field application (orthopedic, cardiovascular system devices, wound management, drug delivery, and others) [7]. Following the carbon source [8], downstream processing contributes to a major share of PHAs’ production cost [9]. Hence, it has been considered a bottleneck in providing a competitive price for PHAs on the market. Furthermore, conventional PHA purification techniques such as solvent extraction, enzymatic and chemical digestion, and others have the downside of not being environmental-friendly enough due to the large amount of volatile and toxic solvent consumption, the disruption and degradation of the PHAs’ natural morphology as well as the high cost [10,11]. Therefore, there is an urgent need for a cost-effective and green strategy to purify and recover PHAs in large-scale. 

Aqueous two-phase extraction (ATPE) is a purification tool that exploits the preferable partitioning of biomaterials in a two-phase system formed due to the salting-out of the polymer by sulfate or phosphate salts and the immiscibility of two structurally different polymers or salts. ATPE works by partially removing the target product from substrates or impurities, thus, minimizing the subsequent downstream processing volume [12]. Thermoseparating-based ATPE is an ATPE technique which utilizes thermoseparating ethylene oxide–propylene oxide (EOPO) random copolymers which have the unique characteristic of decreasing solubility in aqueous solution as the temperature rises. After the first stage of purification using thermoseparating-based ATPE, the aqueous solution of the thermoseparating polymer is heated until it exceeds the threshold temperature and then thermoseparates into two phases, enabling the recovery and recycling of the EOPO [13]. Not only that, thermoseparating-based ATPE make use of mild environment with water content up to 80–90 wt % for bioseparation of sensitive bioproducts [14]. Furthermore, the separation technique employs phase-forming components which are non-toxic and relatively environmental-friendly. Not only has it the ability to handle a large amount of feedstock with a fast processing time, but also the scale-up of ATPE can be predicted reliably without difficulty from laboratory experiment data [15]. Hence, thermoseparating ATPE offers a solution for the demand of a highly efficient as well as cost-effective industrial-scale bioseparation technique for PHAs. On the other hand, the main downside of bioproduct purification using ATPE is the complex mechanism behind the partition of the target product which is influenced by a long range (hydrophobic and electrostatic) and short range (van der Waals) molecular interaction between the bioparticle and the system [16]. In addition, there is a shortage of suitable mathematical models to explain it [17]. As there are many parameters which play their part in bioproduct partitioning by ATPE, this results in a highly complex task for optimization. The conventional method of single factor optimization which optimizes one parameter at a time while keeping all the other variables constant using a trial and error approach [17] is highly unfitting for the purpose of multivariable optimization. This is due to the huge amount of time and labor consumption required with a large number of experiments needed to be conducted, as well as both not guaranteeing the identification of the global optimum and not being able to give insights on interactions between the independent parameters [18]. 

In order to overcome this, statistical design of experiment (DOE) was widely utilized for optimization, modeling, and identification of parameters which govern the bioproducts partition behavior as well as the significant interactions between them by utilizing a minimum amount of experiments [19]. Nevertheless, a thorough literature review showed that the application of DOE on PHAs purification had not yet been described. Thus, a two-level full factorial design was utilized to study the influence of the concentration of potassium phosphate, EOPO, NaCl addition, as well as pH on the partition behavior of PHAs. Mathematical modelling has been used widely as a tool to describe the complex interaction or behavior of a system. One of the applications of modeling is to serve as a prediction model to help researchers to study the outcome of a process without undergoing any experimental procedure. Application of the empirical model is more preferred than the mechanistic model in an area that may be viewed as a highly complex nonlinear process or system, due to its straightforward development. One of the highly regarded empirical modeling tools is feed forward neural network (FFNN). FFNN is commonly used in chemical and process control applications which demonstrate its capability to perform system identification and control for a wide range of dynamic and nonlinear systems [20]. FFNN also provides the flexibility and complexity to approximate nonlinear functions to any desired accuracy by varying the number of layers and hidden neurons in each respective layer [21]. In this work, FFNN was used to develop a model for the PHAs purification process using thermoseparating ATPE. An experimental work was conducted to collect the necessary data. In order to improve the generalization of FFNN, the experimental data is resampled using the Bootstrap resampling method. Therefore, this paper demonstrated two different approaches to model the extraction of PHAs via thermoseparating ATPE.

## 2. Materials and Methods

### 2.1. Materials

*C. necator* H16 strain was kindly provided by Yuan Ze University, Taoyuan, Taiwan. Poly(ethylene glycol-ran-propylene glycol) mono butyl ether (EO:PO:50:50, *M*n ~ 3900), benzoic acid, and methanol were purchased from Sigma-Aldrich (M) Sdn Bhd (Selangor, Malaysia). All the other chemicals used in this study were of analytical grade.

### 2.2. Production of PHAs

The bacterial cells were first cultured on agar plates at 30 °C for 24 h. An inoculum was prepared in 10 mL nutrient broths (8 g/L) by inoculating the medium with a single colony from the cultured agar plate. The culture was then grown aerobically at 30 °C for 24 h. Subsequently, the inoculum (5% *v*/*v*) was transferred to a 250 mL shake flask containing 100 mL operating volume of defined medium supplemented with glycerol of 30 g/L and yeast extract of 2 g/L. The inoculated fermentation medium was then incubated at 30 °C for 72 h at a shaking speed of 100 rpm. The defined medium was composed of Na_2_HPO_4_-7H_2_O, 6.7 g/L; KH_2_PO_4_, 1.5 g/L; (NH_4_)_2_SO_4_, 2.5 g/L; MgSO_4_-7H_2_O, 0.2 g/L; and CaCl_2_, 10 mg/L) and 0.5% *v*/*v* of trace mineral solution (Na_2_EDTA, 6.0 g/L; FeCl_3_-6H_2_O, 0.29 g/L; H_3_BO_3_, 6.84 g/L; MnCl_2_-4H_2_O, 0.86 g/L; ZnCl_2_, 0.06 g/L; CoCl_2_-6H_2_O, 0.026 g/L; and CuSO_4_-5H_2_O, 0.002 g/L).

Without undergoing an extra clarification or filtration step, the cultured broth of *C. necator* H16 was directly subjected to ultrasonic cell disruption with an ultrasonic processor (Hielscher, UP4005) at 30 kHz per cycle for 15 min to obtain intracellular PHAs.

### 2.3. Experimental Design and Statistical Analysis

A two-level full factorial design (2^4^) was carried out to investigate the effects and interactions of important parameters in the thermoseparating-based ATPE of PHAs. Four independent parameters, including potassium phosphate concentration (X_1_), EOPO concentration (X_2_), pH (X_3_), and sodium chloride (X_4_) were taken into account and studied at two widely spaced levels. The low (−) and high (+) levels of the factors were 8 and 15 wt % for potassium phosphate concentration, 10 and 18 wt % for EOPO concentration, 8 and 10 for system pH, and 0 and 100 mM for sodium chloride addition. The reason behind the choice of the levels of EOPO concentration and phosphate concentration is to ensure the formation of a two-phase with a high enough volume ratio so that the “volume-exclusion” effect does not occur. Volume-exclusion occurs when the free space available for target products in the polymer-rich top phase is reduced, causing partitioning to the bottom phase [22]. System pH levels were chosen based on a literature review of PHAs partitioning [23]. Levels of sodium chloride addition were selected following the results of the previous study where 10 mM of salt addition gave recovery yield as high as 90.9%, while 100 mM of salt addition gave recovery yield as low as 53.5% [24]. These four parameters were chosen due to their potential influence and contribution to the partitioning of PHAs by thermoseparating polymer-based ATPE based on a thorough literature review and previous experimental study [24]. The experiments were conducted in a completely random manner to fulfill the requirement of each run being independent of the influence of an unknown effect. Three replicates of the full factorial design experiments were conducted.

### 2.4. Partitioning of PHAs in Thermoseparating ATPE

A 40 g two-phase system was prepared by mixing the phase-forming components which are EOPO and salt (in wt % following the experiment set up) with the crude feedstock. The mixture was then allowed to settle for phase separation. After the stages of equilibration and phase separation, the top phase was carefully sampled using a pipette, while the bottom phase was then sampled through the interface. The samples from both the phases and disrupted biomass were centrifuged at 150 rpm for 15 min and washed with 20 mL of deionized water three times before being left in the oven overnight at 70 °C to be dried. The dried samples from both phases and disrupted biomass were analyzed for PHAs content using gas chromatography (GC). 

### 2.5. Quantification of PHAs by Gas Chromatography (GC) Analysis

For the determination of PHAs content, the GC method of Akaraonye et al. with slight modification was employed [25]. Two mL of chloroform and 2 mL of acidified propanol which contained 15% *v*/*v* of 98 wt % sulphuric acid were added to about 20 mg of dried sample. After incubation at 100 °C for 2 h, the samples were cooled rapidly with running water to ambient temperature and 1 mL of water was then added to the sample to remove the sulfuric acid. The sample was then allowed to settle until separation into organic and aqueous phases. Then, 0.2 μL of the organic phase (bottom phase) which contain butyrate ester dissolved in chloroform was injected into a Clarus 500 gas chromatograph (Perkin Elmer, Waltham, MA, USA) equipped with a DB-WAX capillary column (0.25 mm by 30 m; 0.25 μm film thickness). The initial oven temperature was set at 60 °C and held for 3 min, then increased to 210 °C at a rate of 10 °C/min and held for 3 min at the same temperature. The PHAs content of the injected samples was determined by internal standard calibration using standard PHAs (Sigma-Aldrich) with benzoic acid as internal standard.

### 2.6. Partitioning Behaviors of PHAs 

The partitioning of PHAs can be described by their respective partition coefficients (*K*pa), which is defined as the ratio of PHAs concentration in the top to that in the bottom phase:

*K*pa = Conc_T_/Conc_B_ (1)

where Conc_T_ and Conc_B_ are PHAs concentration of top phase and bottom phase respectively.

The purity % was defined as the ratio between the mass of PHAs quantified by GC and the total mass of dried sample used for the GC analysis:

Purity = (*M*_PHAs_/*M*_Sample_) × 100% (2)

where *M*_PHA_ is the mass of PHAs (g) and *M*_Sample_ is the total mass of dried sample used (g).

The phase volume ratio, *V*_r_ was defined as the ratio between top phase volume and bottom phase volume:

*V*_r_ = *V*_T_/*V*_B_ (3)

where *V*_T_ and *V*_B_ are top and bottom phase volume respectively.

Recovery yield % of PHAs in the top phase was calculated as the ratio between the PHAs mass in the top phase and the initial PHAs mass in the extract:

Recovery yield (%) = (Conc_T_ × *V*_T_)/(Conc_E_ × *V*_E_) (4)

where Conc_T_ and Conc_E_ are the PHAs concentration of top phase and extract, respectively and *V*_E_ is the extracted volume.

Purification factor (PF) was defined as the ratio of the top phase PHAs purity to the initial PHAs purity:

PF = (Top Phase PHAs Purity)/(Initial PHAs Purity)(5)

### 2.7. Neural Network Methodology

**Figure 1:**
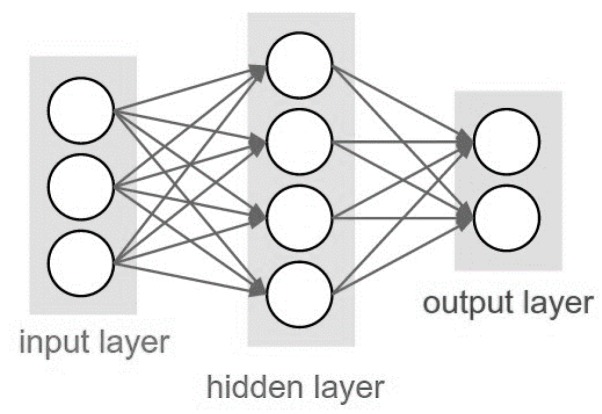
Feed forward neural network (FFNN) model topology with single hidden layer.

**Figure 2:**
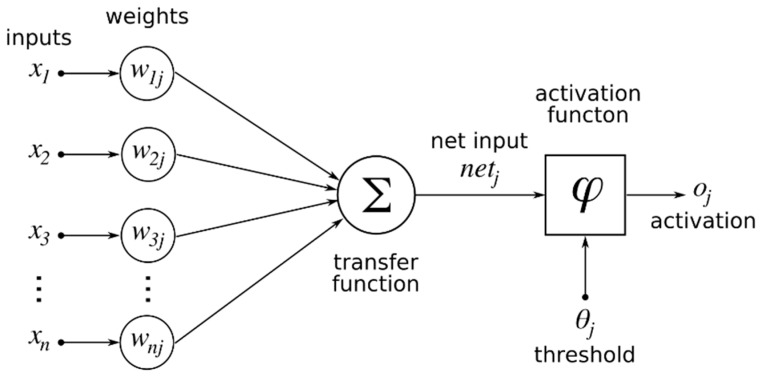
A single node of neural network.

Feed Forward Neural Network (FFNN) is a straightforward type of neural network where the information moves only in one direction (i.e., forward) from the input nodes, through the hidden nodes, and to the output nodes. There are no cycles or loops in the network. The group of nodes in each respective column is called a layer. A typical FFNN with a single hidden layer is shown in Figure 1. The lines connecting the input layer neurons and hidden layer neurons represent the network weights. The hidden neuron sums up the corresponding weight from all input connections. The weighted summation is then passed through an activation function in the hidden layer. The activation function such as sigmoid, gives the FFNN model the ability to select the appropriate information to be passed on to the next neuron. A basic node or computing element for the FFNN model is shown in Figure 2. A threshold or bias is generally used to regulate the network performances. In order to generalize the relationship between the input and output, the FFNN model is trained using predetermined data. During this training, the FFNN model learns the behavior of the model by adjusting its weights and biases. The training process is usually done using a backpropagation algorithm to minimize certain “cost function” such as mean squared error (MSE).

**Figure 3:**
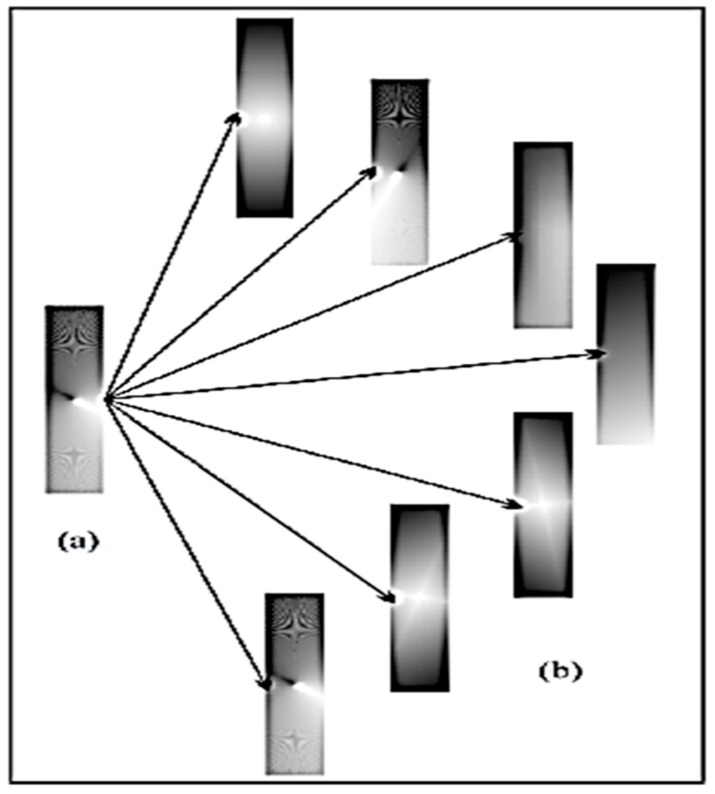
Analogy of bootstrap resampling technique: (**a**) Data distribution in original dataset; (**b**) data distribution in new datasets after resampling [29].

In this work, a set of four input and three output parameters were selected to develop the synthesis model of PHAs. The selection of the input-output of the model is the same as in the experimental work, which was carried out to determine the significant parameters in the synthesis procedure. Since there is more input than output, the use of a single hidden layer in the FFNN topology suffices [26]. The choice of one hidden layer is usually sufficient for the purpose of approximation of continuous nonlinear function as more hidden layers may cause over-fitting [21]. However, the amount of available experimental data is limited and this can hinder the FFNN model in being properly generalized during its training process. In order to generate and replicate more data for the FFNN training, the bootstrap resampling method is used [27]. The bootstrap method uses randomization technique to rearrange and resample the original data into a new larger dataset. This technique has proven to improve the generalization and robustness of the neural network model [28]. A descriptive overview of how data is resampled and redistributed by using this technique is illustrated in Figure 3. In the original dataset, the data are distributed as noted by the color intensity. After resampling, the new datasets have a randomized distribution with replacement of the original data (refer the color intensity of the new datasets). In this study, the bootstrap technique was used to produce 160 data points from the original 16 experimental data points. This new dataset was divided randomly into training (60%), validation (20%), and testing dataset (20%).

The performance of the FFNN was measured using the mean squared error (MSE), root mean squared error (RMSE), and correlation of determination (*R*^2^). In this work, the FFNN was trained using the Levenberg-Marquardt backpropagation technique. This technique is well known to produce FFNN with good generalization and fast convergence. The FFNN is trained iteratively using different numbers of hidden neurons in order to acquire the best model with the lowest MSE and RMSE value with *R*^2^ near to one [30]. All of the simulation work regarding neural network modeling and analysis was performed using Matlab software.

## 3. Results and Discussion

### 3.1. Statistical Experimental Result

In ATPE, biomolecules have a complex partitioning behavior which is influenced by the charge, molecular size, electrochemical properties, and hydrophobicity of the proteins. The partitioning of biomolecules can be manipulated by manipulating factors like the concentration of phase-forming components, pH, and salt additions. A two-level full factorial design (2^4^) was conducted to investigate the influence and interaction of four variables, which were potassium phosphate concentration (X_1_), EOPO concentration (X_2_), pH (X_3_), and NaCl addition (X_4_) in PHAs partition by thermoseparating-based ATPE. Full factorial designs and the responses are presented in Table 1. It can be seen that there is a wide variation of *K*pa (0.402–16.547) and recovery yield (16.8%–93.9%) which is due to the intended variation in the factor combinations and this revealed the significance of optimization in achieving better recovery and purity. From the results in Table 1, it can be seen that run 6 has the highest *K*pa (16.5) and yield (93.9%), while run 10 has the highest PF with the value of 1.54. Utilizing Design Expert software, the analysis of variance (ANOVA) was performed to verify the validity of the models, evaluate the statistical significance of all factors, and determine the influence of these factors on the response variables. These models consisted of four main effects, six two-parameter interactions, and four three-parameter interactions, while the last which is one four-parameter interaction was given the assumption of being negligible due to hierarchical reasons [29].

#### 3.1.1. Effect on the “Yield”

From a practical point of view, efficient purification and extraction of ATPE require the maximum recovery of a target product with the purity as high as possible. From Table 1, the results demonstrated that the maximum yield was achieved in run 6 with a value of 93.9%, while the minimum was obtained in run 9 with a value of 16.8%. The full statistical results for response variable “Yield” are shown in Table 2. On the basis of the result, the *f*-value of 1007.69 with low probability value (Prob>F = 0.0247) indicates that the the overall regression model was significant with 95% confidence. The value of the coefficient of determination, *R*^2^, can be utilized to assess the ratio of total variation ascribed to each fit. With a value always between 0 and 1, *R*^2^ larger than 0.75 shows a good fit of the model to the response variable [31], while a value larger than 0.9 is very satisfying in the DOE for the bioprocess [22]. For recovery yield, the high value of *R*^2^, which is 0.9999 demonstrates a good response between the model and the experimental results. This also indicates that the interrelationship between the independent variable can be satisfactorily represented by the model with only less than 0.01% of total variations not able to be explained by the model. The adjusted coefficient of determination, *R*^2^_adj_ can be used to measure the accuracy of a model for the response variable [6]. The *R*^2^_adj_ of 0.999 was in a good agreement with the predicted *R*^2^, *R*^2^_pred_ of 0.982, which shows that the predicted values are compatible with the experimental results. 

Values of “Prob>F” less than 0.05 reveal that the model terms are statistically significant model terms, while values larger than 0.10 show that these are insignificant model terms [19]. For recovery yield, the regression analysis of the experimental design indicated that the linear model terms (X_1_ and X_2_) and interactive model term (X_1_X_2_) were the significant model terms. By discarding and pooling all statistically insignificant model terms (Prob>F more than 0.05) into the error term and using only significant model terms, the new reduced model was obtained for response variable “Yield”. Using ANOVA, the statistical analysis demonstrated that the reduced model was significant at a confidence level of 95% with the p-value much lower than 0.05 (Prob>F less than 0.0001). By taking into account the significant linear model terms and interactions, the regression analysis of Yield data provided the following first-order model:

Y = 45.21 + 21.39 X_1_ + 12.52 X_2_ + 10.56 X_1_X_2_ (6)

Generally, the contribution of the model terms to the response variables can be evaluated utilizing the degree of the corresponding coefficients of the linear regression equations [32]. From the regression equation, it can be observed that potassium phosphate concentration (X_1_) had the strongest positive effect on recovery yield. As shown in the experimental design results, the recovery yield is generally higher at high (+) level of X_1_ where the yield achieves as high as 93.9% for run 6. In the system of higher phosphate concentrations, the strengthened “salting-out” effect reduces the solubility of PHA in the salt-rich bottom phase, by promoting aggregation and hydrophobic interaction. This, in turn, directs PHA partition to the polymer-rich top phase which has lower salt concentration, facilitating the extraction of PHAs to the EOPO-rich phase [33]. This is in accordance with other works in the partitioning of others biomolecules by ATPE, such as collagenase from *Penicillium aurantiogriseum* [29] and other bioproducts as well. More importantly, it is worth mentioning that the highest yield obtained in the current study is significantly higher than that of the literature which utilized the PEG/phosphate system (40% to 50%) [3].

In addition to X_1_, the regression equation also suggested that EOPO concentration (X_2_) is a significant positive parameter for recovery yield as well. There are two main forces dominating in the polymer-rich phase, which are the “volume-exclusion” effect and the hydrophobic interaction. As the “volume-exclusion” effect no longer in the picture as mentioned above, the increasingly stronger hydrophobic interaction between polymer-rich phase and PHAs molecules due to increasing concentration of thermoseparating polymers causes PHAs partition preferably to the top phase [32]. Several studies on ATPE also showed similar results, such as for lysozyme [16] and other bioproducts. Another essential point is that the recovery yield was also positively affected by the synergistic positive interaction between phosphate and EOPO concentration (X_1_X_2_). The simultaneous rise in the level of both parameters had a stronger impact on the increment of recovery yield than the expected add up of those of the individual parameters. The combined effect can be observed in run 6, 7, 10, and 11 where the yield can achieve a high value of at least 85% when both phosphate and EOPO concentration are at high (+) levels.

Supported by a literature review [3], the recovery yield remains constant in the selected range for pH, thus, it is not a significant model term with 95% of confidence. Though, with Prob>F of 0.0519, pH still has a positive impact on the recovery yield, agreeing with the results of Table 1 (with the exception of run 5 and 15 as well as run 7 and 10 due to the negative contribution from other parameters). In this respect, it should not be forgotten that system pH serves an important role in thermoseparating ATPE as it influences partitioning of the bioproduct by modifying the solute charge [29]. Therefore, PHA partitioning is preferable to be performed at a more basic pH [3], although it is still worth mentioning that extreme pH will cause degradation to PHA granule morphology. 

Because of the diverse affinity of ions for the different phases where anions give a stronger effect compared to cations, the addition of co-solutes like salts into a two-phase system serve as “counter-ions” which promote the partition of biomolecules to the desired phase. The reason the salt addition parameter is not significant at the 5% level might be due to the high content of multiple salts (approximately 80 to 175 g/L) in the PHAs crude extract used and thus, the NaCl addition does not have a dominating effect. Nevertheless, very slight salt addition with Prob>F of 0.0717 gives a negative influence on recovery yield, as illustrated in the results of Table 1 (except for run 10). Previous ATPE works on clavulanic acid from the fermentation broth of *Streptomyces clavuligerus* [22] also illustrated similar results. Thus, according to the full factorial design, the addition of NaCl as co-solute should be avoided for maximum recovery of PHAs. To sum up, PHAs yield as high as 93.9% can be obtained at a high concentration of both EOPO 3900 (18 wt %) and phosphate salt (15 wt %) as well as the condition of basic pH without any addition of NaCl.

#### 3.1.2. Effect on the “Partition Coefficient”

Generally employed to assess the effectiveness of biomolecule separation by ATPE, the extreme value of the partition coefficient (*K*pa) signifies the effective extraction of target biomolecules from systems, while a value close to unity (1) indicates an almost equal partition between both phases which is undesirable in ATPE. For thermoseparating ATPE, the partitioning of target products to the polymer-rich top phase (*K*pa >1) is highly desirable due to the exclusive recovery of bioparticles in the water-rich top phase during the secondary ATPE. As shown in Table 1, the maximum *K*pa (16.547) was achieved in run 6, while run 15 gave the minimum *K*pa (0.369). The highest partition coefficient achieved in this study is comparable to that of the literature which utilizes the PEG/phosphate system (*K*pa = 4 to 15) [8]. The statistical results for the reduced model of response variable “*K*pa” are presented in Table 3. Statistical analysis of *K*pa indicated that three out of the four linear model terms, which are X_1_, X_3,_ and X_4_ were statistically significant with 95% of confidence and had a significant influence on *K*pa. For this response variable, EOPO concentration only gives a slight but positive effect on the intended selection of X_2_ levels to prevent the “volume-exclusion” effect. Not only that, some of the interactive model terms (X_1_X_2_, X_2_X_3_ and X_2_X_3_X_4_) were significant at 95% of confidence level as well. The reduced model can be described by the following regression equation:

1/*K*pa^0.5^ = 0.95 − 0.43 X_1_ − 0.057 X_3_ + 0.046 X_4_ − 0.18 X_1_X_2_ + 0.054 X_2_X_3_ + 0.033 X_2_X_3_X_4_ (7)

It is worth mentioning that the empirical relationship between the *K*pa and the test variable is better characterized using an inverse square root function instead of a normal linear function due to the significant deviation from normality demonstrated. The *f*-value of 221.5 and *p*-value at a level below 0.0001 demonstrated a very high significance for the regression model and confirmed the adequacy of the reduced model. With the high value of *R*^2^ (0.997), it shows that the real relationship between the response variable and independent parameters is adequately represented by the model. This indicates that 99.7% of the variability in response could be explained by the model. The close values of *R*^2^_adj_ and *R*^2^_pred_ which are 0.992 and 0.979 respectively demonstrate a good degree of correlation between the theoretical values predicted by the reduced model equation and experimental responses.

Similar to the response variable “yield”, the most significant and positive influence on the response variable “*K*pa” at a confidence level of 95% was that exerted by the potassium phosphate concentration (X_1_). As shown in Table 1, only *K*pa for a high level (i.e., 15 wt %) of X_1_ gives a value larger than 1 (ranging from the lowest of 1.3 to the highest of 16.5). The same result was also reported in the works of partitioning of collagenase [29] and lysozyme [16]. Also, a positive interaction effect between X_1_ and X_2_ was observed, revealing a synergism between the two variables. Therefore, higher *K*pa will be achieved with a high level of both potassium phosphate and EOPO concentration, which can be seen especially in run 6 and 10 (with *K*pa higher than 12). 

For salt addition, in agreement with “Yield” response this should be completely avoided to achieve high *K*pa. Agreeing with Divyashree and his co-workers who reported that significant increase in *K*pa can be observed after pH 8 [23], the results of Table 1 demonstrated that high *K*pa can be obtained at a higher pH value. This is also observed in the partitioning of fibrinolytic proteases from *Streptomyces* sp. DPUA1576 in the PEG/phosphate system [22]. However, the simultaneous increase in the level of EOPO concentration and pH lowers the value of *K*pa unless there is a dominating positive influence from X_1_ as in run 6. 

For this response variable, there were some significant two- and three-parameter interactions involving every parameter which can be observed. The overall influence of a given parameter on this response variable is affected by the levels of the other parameters. Thus, this makes the estimation of these effects on *K*pa very complicated due to the strong influence of one of the parameters on the interaction to the others. Still, it can be concluded that both high salt and EOPO concentration with basic pH and no addition of cosolute will contribute to high *K*pa in PHAs partitioning.

#### 3.1.3. Effect on the “Purification Factor”

Serving as the primary purification tool of PHAs, the purification factor (PF) is used to define the purification efficiency of thermoseparating ATPE. In contradiction to the wide variation of *K*pa and yield, the PFs obtained in the factorial design were in a smaller range where an average value of 1.235 with the fluctuation of 0.3 was achieved for most runs (with the exception of run 9 which had a value of 0.67). Based on the statistical results for reduced model of response variable of “PF” illustrated in Table 4, the reduced model was significant at a confidence level of 95% with f-value of 42.6 and probability value much lower than 0.05 (Prob>F less than 0.0001). With a value of 0.966 for *R*^2^, this indicated that 96.6% of the experimental data was compatible with the predicted data from the model. The value of *R*^2^_adj_ was calculated to be 0.943, close to that *R*^2^_pred_ (0.893), which demonstrates a high degree of correlation between the predicted and observed values. The model can be adequately utilized to predict the data within the range of variables studied. The reduced model can be described by the following first-order model:

PF = 1.15 + 0.17 X_1_ + 0.11 X_2_ − 0.067 X_1_X_2_ − 0.062 X_2_X_3_ − 0.077 X_1_X_3_X_4_ (8)

The regression analysis of PF indicated that the X_1_ and X_2_ were significant with 95% of confidence among the investigated independent parameters as shown in the equation. Other than that, the interactions between independent parameters such as X_1_X_2_, X_2_X_3,_ and X_1_X_3_X_4_ also play significant roles in the purification of PHAs by thermoseparating ATPE. As expected of the positive main effect of phosphate concentration, a dramatic increase of PF values can be observed at the elevated levels of this parameter, especially shown in run 10 and 11 with PF greater than 1.5. This was also demonstrated in the partition of collagenase from *Penicillium aurantiogriseum* by Lima and his colleagues with improving purification when utilizing higher phosphate concentration [29]. Similarly, PF was found to be positively correlated with the EOPO concentration (X_2_) as well. This trend is congruent with the purification of other bioproducts utilizing ATPE as reported in the literature. For example, the purification factor of α-amylase from *Aspergillus oryzae* was increased by threefold at the highest level of PEG concentration (20 wt %) [31]. Study on isolation of lysozyme from crude hen egg white has reported a high PF value was achieved at high polymer concentration as well [32]. Despite that, the simultaneous increase of X_1_ and X_2_, as well as X_2_ and X_3_, has a negative impact on PF. To summarize, PF is the most complicated response variable to be optimized due to the complicated positive contributions from the linear model terms combined with the negative contributions from the interactions between them.

### 3.2. Feed Forward Neural Network (FFNN) Model Results

**Figure 4:**
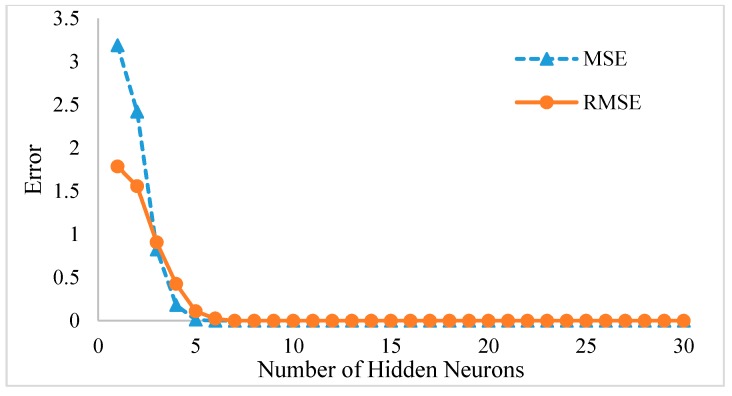
FFNN error results with a different number of hidden nodes.

After successfully determining the significant parameters in the PHAs synthesis process, this research continued by developing a simulation model of the process by using Feed Forward Neural Network (FFNN). In this work, the FFNN model utilizes the experimental input and output data of the process in order to generalize its relationship. In order to develop a reliable FFNN model, the model needs to examine the best number of hidden neurons in its model. Figure 4 shows the validation results of the model based on a different number of hidden neurons. The number of hidden neurons tested here were varied from 1 to 30. Based on the figure, it can be observed that the error from both MSE and RMSE starts to decrease until it stays steady at the model with 10 hidden neurons. This shows that the FFNN model can produce good results after 10 hidden neurons. In order to reaffirm the results, each hidden neuron was trained five times and the one with the lowest error was selected. Based on the training results, FFNN with 30 hidden neurons produces the best results with MSE = 1.04 × 10^−17^, RMSE = 3.22 × 10^−9^ and *R*^2^ = 1. The excellent results are assumed due to the application of the resampling method. By providing the FFNN model with a larger set of data, the network was able to generalize properly. However, due to the lack of foreign or unseen data, the possible effect of network overfitting could not be tested.

**Figure 5:**
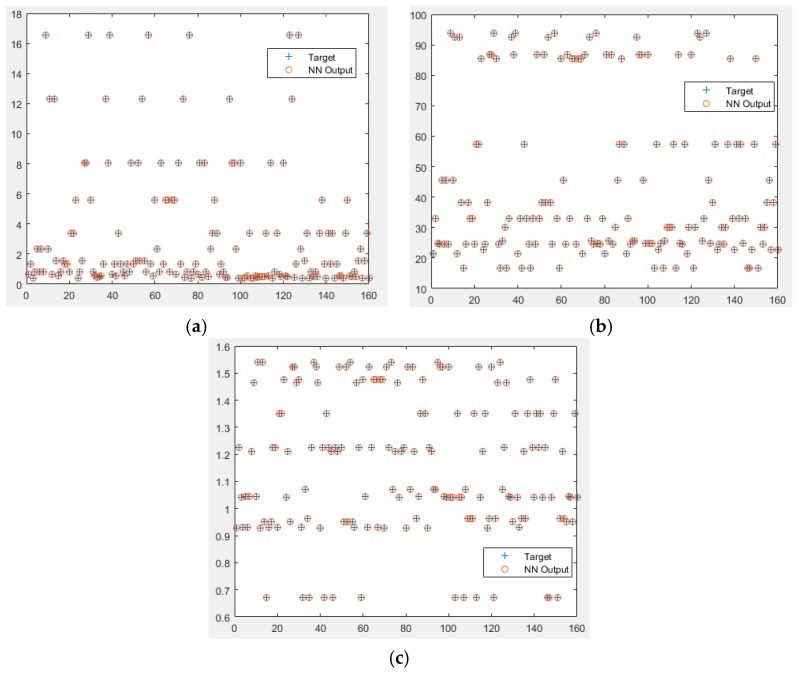
Validation results for Output 1 Partition Coefficients (*K*pa) (**a**); Output 2 Recovery Yield (%) (**b**); and Output 3 Purification Factor (PF) (**c**).

Figure 5 shows the comparison of the FFNN model final performance (NN Output) with the resample experimental dataset outputs (Target). Since the model has three outputs with different scales, the comparison was done individually. Based on overall observation, the FFNN model managed to estimate all the points from the resample dataset with good accuracy. This shows that the FFNN model has successfully encapsulated the behavior of the important input-output parameters in thermoseparating-based ATPE of PHAs. Therefore, this FFNN model can be used to simulate the PHAs synthesis process without conducting any experimental work.

## 4. Conclusions

This work studied a statistical design method to isolate and partially purify PHAs from the fermentation broth of *C. necator* utilizing thermoseparating ATPE. The results of two-level full factorial models on response variables of yield, partition coefficient, and purification factor demonstrated that this strategy can recover PHAs effectively with advantages over the conventional methods. For all the responses, the most influencing factors were the concentration of phosphate salts and EOPO as not only did they provide significant impacts on most of the responses, but the synergistic interaction between the two models terms demonstrated big influence as well. The highest partition coefficient (16.6) and yield (93.9%) can be obtained at the conditions of 15 wt % phosphate concentration, 18 wt % EOPO concentration, and pH 10 without the addition of NaCl. On the other hand, the highest purification factor (1.54) can be achieved at the same concentration of phosphate and EOPO, but at pH 8 and with 100 mM addition of salt as co solutes. Further studies can be done on optimizing the purification conditions of PHAs using the significant parameters obtained. This opens promising standpoints for utilizing thermoseparating ATPE as the primary step in the isolation and purification of PHAs from fermented broth. Furthermore, the application of Feed Forward Neural Network (FFNN) was also tested on the PHAs synthesis process. Due to limited experimental data, the bootstrap resampling method was used to generate more data for the FFNN modeling process. Application of this resampling method proved to be satisfactory based on the final performance of the FFNN. Based on the final results, FFNN has proven its capability to simulate the synthesis process. This model has several applications in the future such as for soft sensors and process optimization.

## Figures and Tables

**Table 1:** Experimental design and results of response variables.

Run	X_1_ (wt %)	X_2_ (wt %)	X_3_	X_4_ (mM)	*K*pa	Yield (%)	PF (fold)
1	8	10	10	0	0.814	24.6	0.93
2	8	10	8	0	0.646	21.4	0.93
3	15	10	10	0	3.371	57.4	1.35
4	8	18	8	0	0.431	25.5	1.07
5	8	18	8	100	0.402	24.8	1.04
6	15	18	10	0	16.547	93.9	1.46
7	15	18	10	100	5.585	85.5	1.48
8	15	10	8	0	1.554	38.3	0.95
9	8	10	8	100	0.525	16.8	0.67
10	15	18	8	100	12.291	92.5	1.54
11	15	18	8	0	8.047	86.8	1.52
12	15	10	8	100	1.314	32.9	1.22
13	8	18	10	0	0.512	30.1	0.96
14	15	10	10	100	2.322	45.5	1.04
15	8	18	10	100	0.369	22.7	1.04
16	8	10	10	100	0.817	24.6	1.21

Where potassium phosphate concentration is X_1_, EOPO concentration is X_2_, pH is X_3_ and NaCl addition is X_4_ and PF is purification factor.

**Table 2:** Full statistical result of ANOVA for response variable “Yield”.

Source	Sum of squares	Degree of freedom	Mean square	F value	Prob>F
Model	12070.8	14	862.2	1007.687	0.0247
X_1_	7323.1	1	7323.1	8558.750	0.0069
X_2_	2507.5	1	2507.5	2930.613	0.0118
X_3_	128.3	1	128.3	149.897	0.0519
X_4_	66.8	1	66.8	78.107	0.0717
X_1_X_2_	1783.0	1	1783.0	2083.799	0.0139
X_1_X_3_	20.9	1	20.9	24.462	0.1270
X_1_X_4_	3.3	1	3.3	3.893	0.2986
X_2_X_3_	100.5	1	100.5	117.459	0.0586
X_2_X_4_	7.7	1	7.7	9.000	0.2048
X_3_X_4_	32.2	1	32.2	37.640	0.1029
X_1_X_2_X_3_	33.4	1	33.4	38.978	0.1011
X_1_X_2_X_4_	20.5	1	20.5	23.931	0.1284
X_1_X_3_X_4_	21.4	1	21.4	25.000	0.1257
X_2_X_3_X_4_	22.3	1	22.3	26.093	0.1231
Residual	0.9	1	0.9		
Cor Total	12071.7	15			
*R*^2^	0.9999				
*R*^2^_adj_	0.999				
*R*^2^_pred_	0.982				

**Table 3:** Statistical result of ANOVA for reduced model of response variable “*K*pa”.

Source	Sum of squares	Degree of freedom	Mean square	F value	Prob>F
Model	3.568	9	0.396	221.5	<0.0001
X_1_	2.891	1	2.891	1615.4	<0.0001
X_3_	0.052	1	0.0516	28.8	0.0015
X_4_	0.033	1	0.0332	18.5	0.0057
X_1_X_2_	0.516	1	0.516	288.5	<0.0001
X_2_X_3_	0.046	1	0.0460	25.7	0.0022
X_2_X_3_X_4_	0.018	1	0.0179	10.0	0.0265
Residual	0.011	6	0.0018		
Cor Total	3.579	15			
*R*^2^	0.997				
*R*^2^_adj_	0.992				
*R*^2^_pred_	0.979				

**Table 4:** Statistical result of ANOVA for reduced model of the response variable “purification factor (PF)”.

Source	Sum of squares	Degree of freedom	Mean square	F value	Prob>F
Model	0.955	6	0.159	42.6	<0.0001
X_1_	0.459	1	0.459	122.9	<0.0001
X_2_	0.205	1	0.205	54.8	<0.0001
X_1_X_2_	0.072	1	0.071556	19.2	0.0018
X_2_X_3_	0.061	1	0.061256	16.4	0.0029
X_1_X_3_X_4_	0.095	1	0.094556	25.3	0.0007
X_1_X_2_X_3_X_4_	0.064	1	0.063756	17.1	0.0026
Residual	0.034	9	0.003734		
Cor Total	0.988	15			
*R*^2^	0.966				
*R*^2^_adj_	0.943				
*R*^2^_pred_	0.893				
